# Leukemia Cutis Associated with Secondary Plasma Cell Leukemia

**DOI:** 10.7759/cureus.1235

**Published:** 2017-05-09

**Authors:** Nicole C DeMartinis, Megan M Brown, Brian R Hinds, Philip R Cohen

**Affiliations:** 1 School of Medicine, University of California, San Diego; 2 Department of Dermatology, University of California, San Diego

**Keywords:** cutaneous plasmacytomas, leukemia cutis, plasma cell leukemia cutis, plasma cell leukemia nodules, secondary cutaneous plasmacytoma

## Abstract

Plasma cell leukemia is an uncommon, aggressive variant of leukemia that may occur de novo or in association with multiple myeloma. Leukemia cutis is the cutaneous manifestation of leukemia, and indicates an infiltration of the skin by malignant leukocytes or their precursors. Plasma cell leukemia cutis is a rare clinical presentation of leukemia. We present a man who developed plasma cell leukemia cutis in association with multiple myeloma. Cutaneous nodules developed on his arms and legs 50 days following an autologous stem cell transplant. Histopathologic examination showed CD138-positive nodular aggregates of atypical plasma cells with kappa light chain restriction, similar to the phenotype of his myeloma. In spite of systemic treatment of his underlying disease, he died 25 days after the presentation of leukemia cutis. Pub-Med was searched for the following terms: cutaneous plasmacytomas, leukemia cutis, plasma cell leukemia nodules, plasma cell leukemia cutis, and secondary cutaneous plasmacytoma. Papers were reviewed and appropriate references evaluated. Leukemia cutis in plasma cell leukemia patients is an infrequent occurrence. New skin lesions in patients with plasma cell leukemia should be biopsied for pathology and for tissue cultures to evaluate for cancer or infection, respectively. The diagnosis plasma cell leukemia cutis is associated with a very poor prognosis.

## Introduction

Plasma cell leukemia is an uncommon variant of leukemia that may occur de novo or in association with multiple myeloma. It is considered the most aggressive plasma cell disorder and is characterized by a proliferation of malignant plasma cells in the blood and bone marrow [1]. Plasma cell leukemia cutis is a rare clinical phenomenon with a poor prognosis [2]. We present a man who developed numerous eruptive cutaneous nodules in association with multiple myeloma.

## Case presentation

A 62-year-old man presented to the hospital for evaluation of progressive vision loss and back pain. His past medical history was significant for plasma cell leukemia-multiple myeloma since September 2015. His treatment regimen was as follows: VCD (bortezomib, cyclophosphamide, and dexamethasone), RVD (lenalidomide, bortezomib, and dexamethasone), Kyprolis-dex (carfilzomib and dexamethasone), pomalyst (pomalidomide), hyper CVAD (cyclophosphamide, vincristine, doxorubicin and dexamethasone) and autologous stem cell transplant.

**Figure 1:**
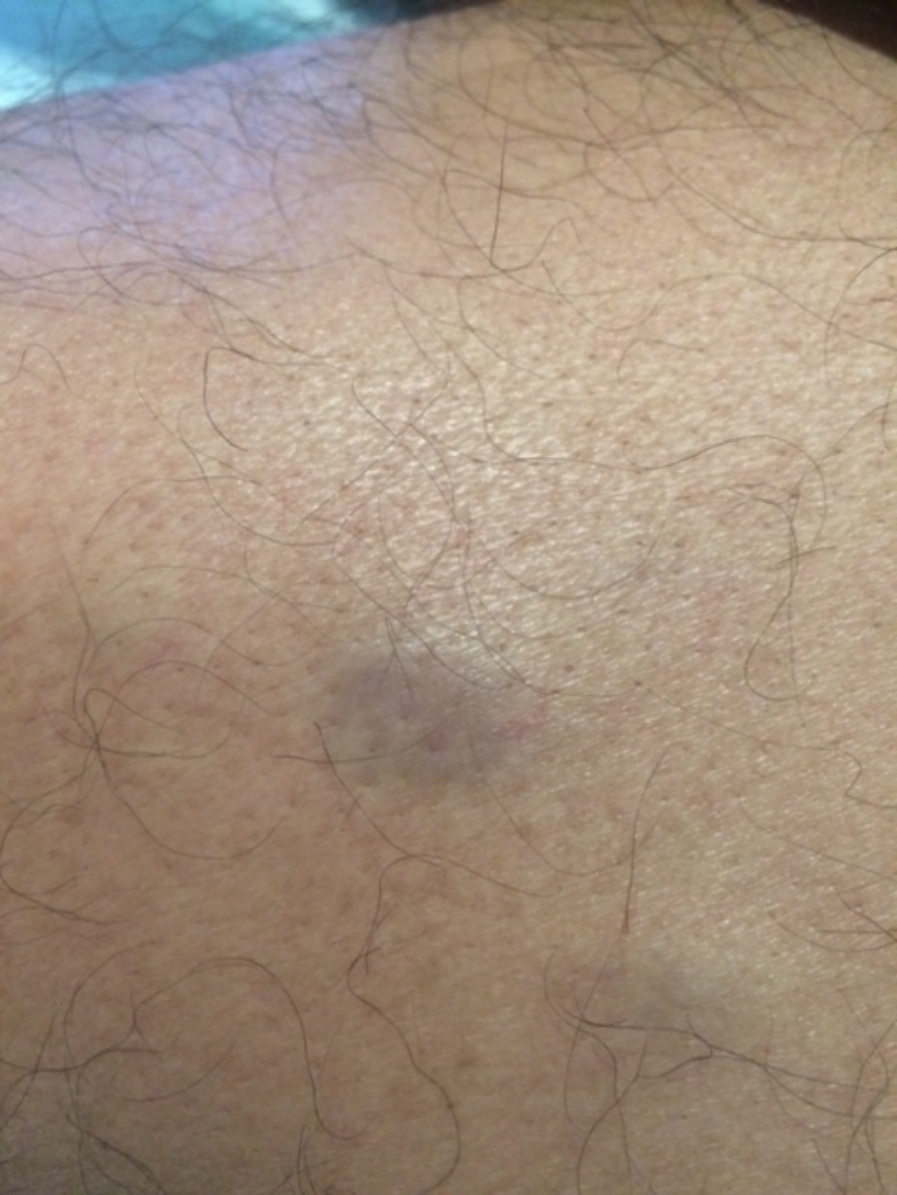
Nodular lesions of plasma cell leukemia cutis on the proximal arm of a man with plasma cell leukemia-myeloma A single erythematous, non-tender nodule of leukemia cutis on the proximal arm.

**Figure 2:**
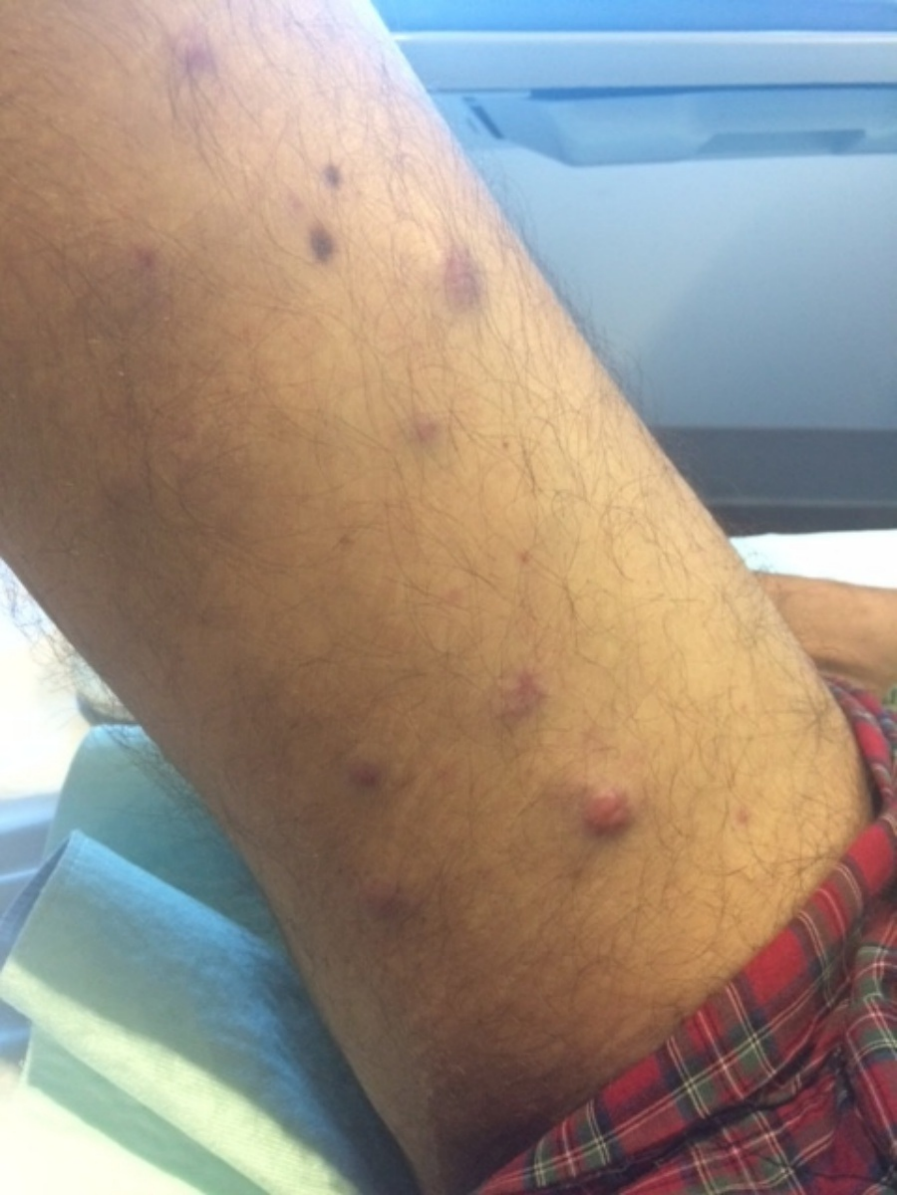
Plasma cell leukemia cutis presenting on the leg of a 62-year-old man with plasma cell leukemia-myeloma Distant view of the proximal right leg showing multiple erythematous, non-tender nodules.

**Figure 3:**
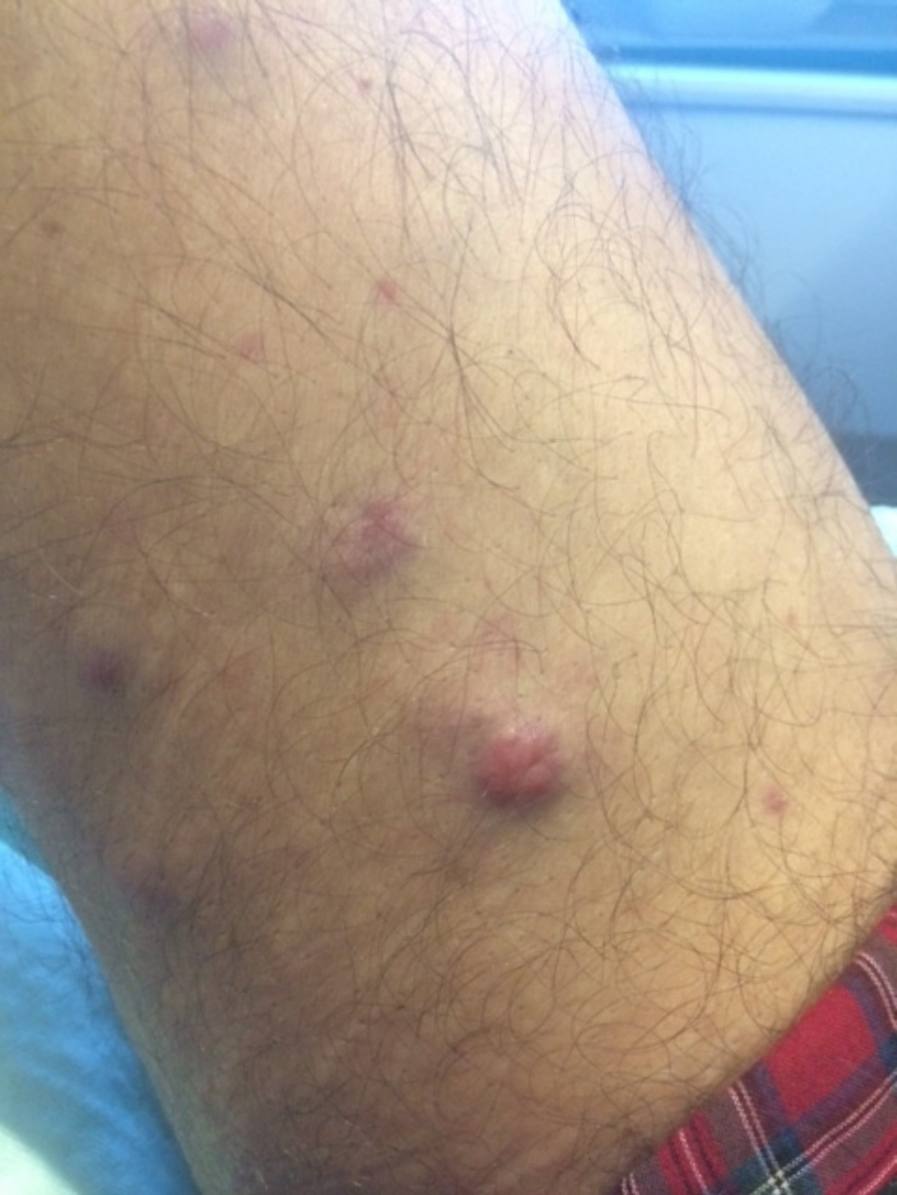
Plasma cell leukemia cutis presenting as cutaneous nodules on the leg Closer view of the proximal right leg showing multiple erythematous, non-tender nodules of leukemia cutis.

**Figure 4:**
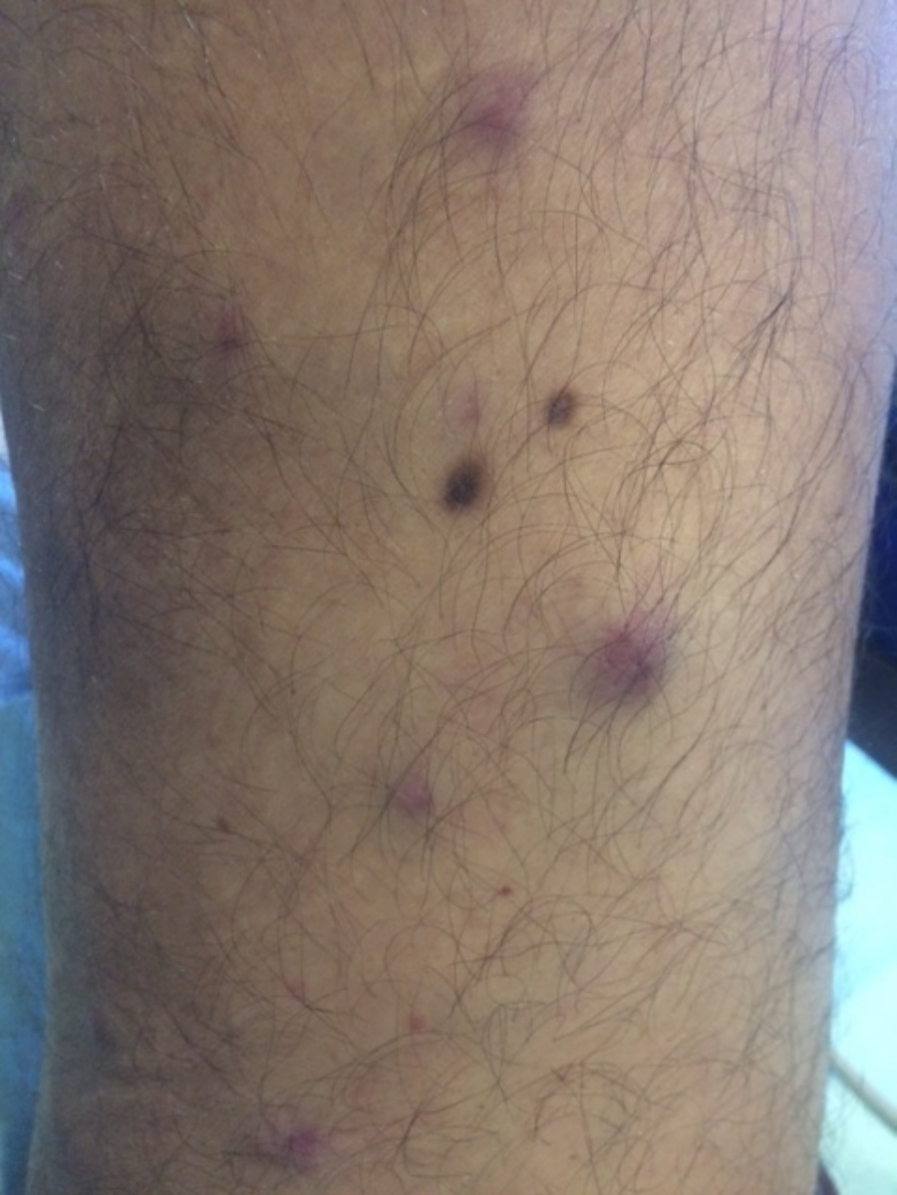
Nodular lesions of plasma cell leukemia cutis on the distal leg of a man with plasma cell leukemia-myeloma Several erythematous, non-tender nodules of leukemia cutis on the distal right leg.

He developed asymptomatic cutaneous nodules on the arms and legs 50 days after stem cell transplant. Cutaneous examination revealed erythematous, firm, non-tender, and smooth nodules, which ranged from 8 mm to 2 cm in diameter on the proximal upper arms (Figure 1) and bilateral lower extremities (Figures 2-4). One nodule was on each proximal arm, 10 were on the right upper leg, and two were on the left upper leg.

His laboratory studies from 11/14/2016 showed a total leukocyte count of 3.1 × 10^9^/L with 45% polymorphonuclear cells and 30% lymphocytes. His hemoglobin was 8.1 gm/dL and the platelet count was 62,000. Monoclonal kappa light chain bands were noted in the blood and urine. Biochemical parameters were: serum creatinine 0.66 mg/dL, serum calcium 8.3 mg/dL, total serum proteins 6.3 g/dL, and alkaline phosphatase 36 U/L. His serum bilirubin and liver enzymes were within normal limits.

Magnetic resonance imaging of his brain (11/11/2016) and spine (11/13/2016) showed leukemic involvement of the brain and lumbar intrathecal space. The patient’s cerebrospinal fluid was found to have large, immature plasmacytoid cells. Flow cytometry revealed clonal, kappa-restricted plasma cells.

**Figure 5:**
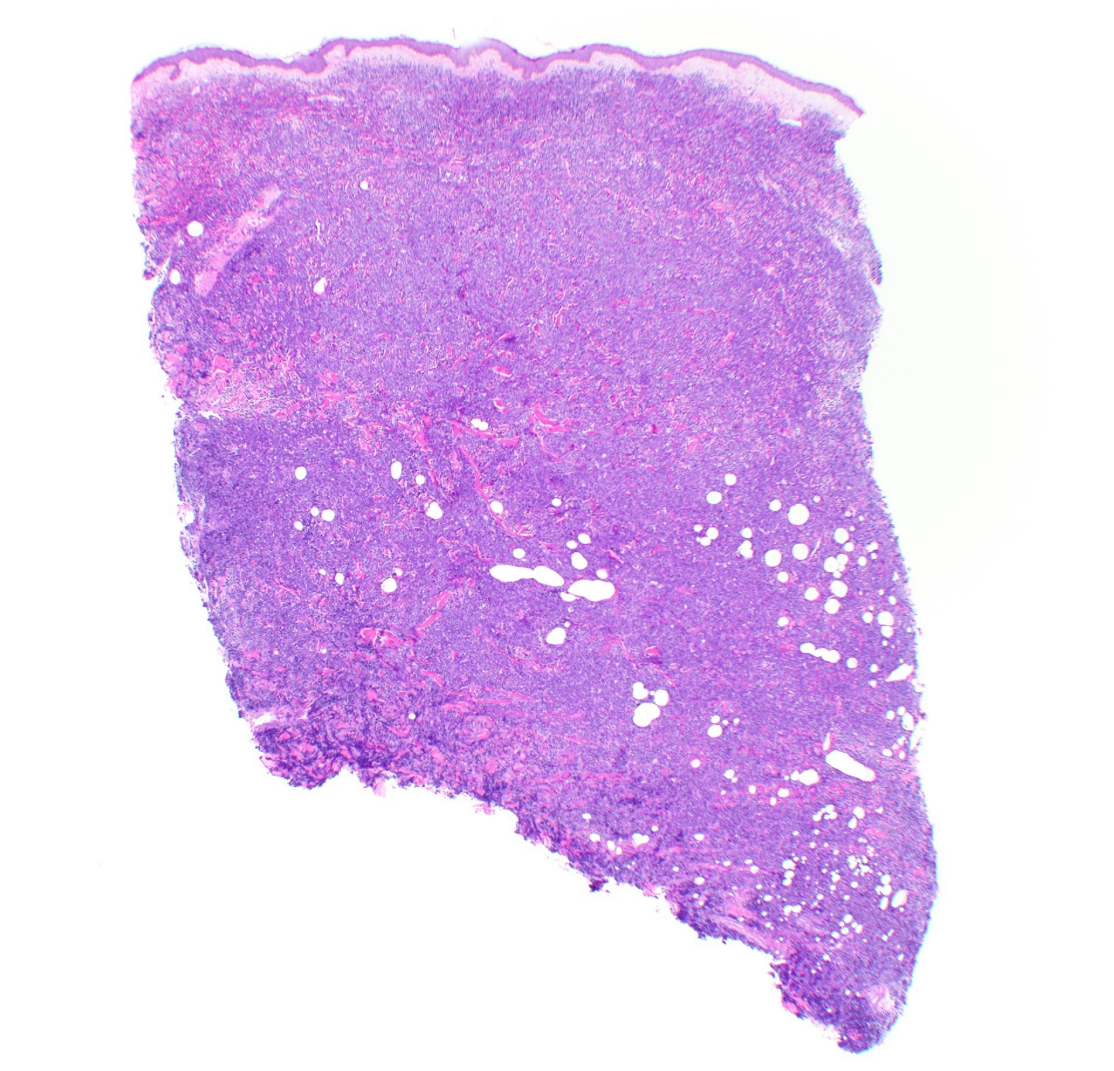
Hematoxylin and eosin stain of skin biopsy of cutaneous nodule from right upper leg Scanning magnification demonstrates non-epidermotropic sheets of lymphoid cells in pan-dermal array extending into the subcutaneous tissue (20x magnification).

**Figure 6:**
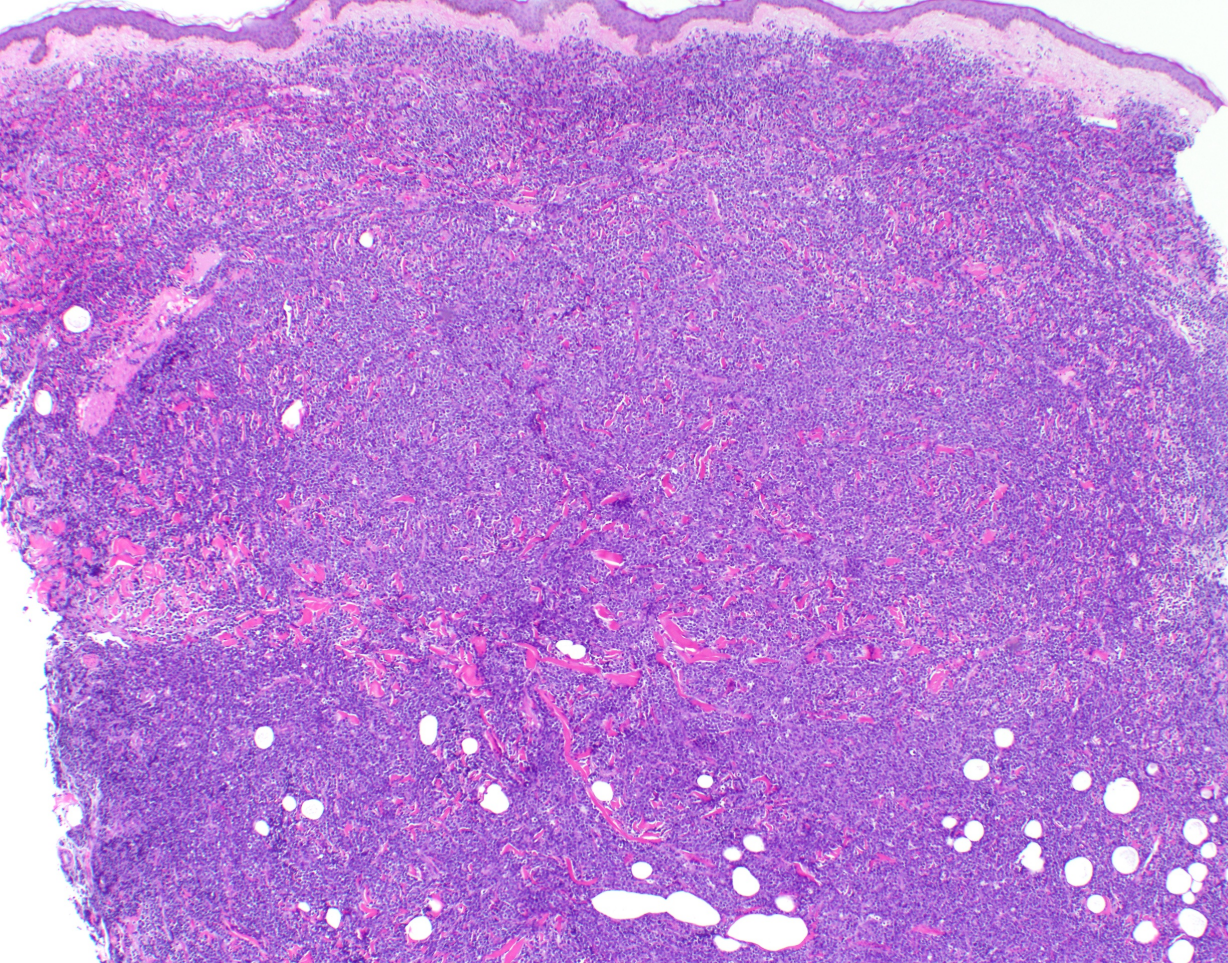
Hematoxylin and eosin stain of skin biopsy of cutaneous nodule from right upper leg A grenz zone is evident along the perijunctional zone and adnexal structures have been resorbed by the atypical lymphoid infiltrate (40x magnification).

**Figure 7:**
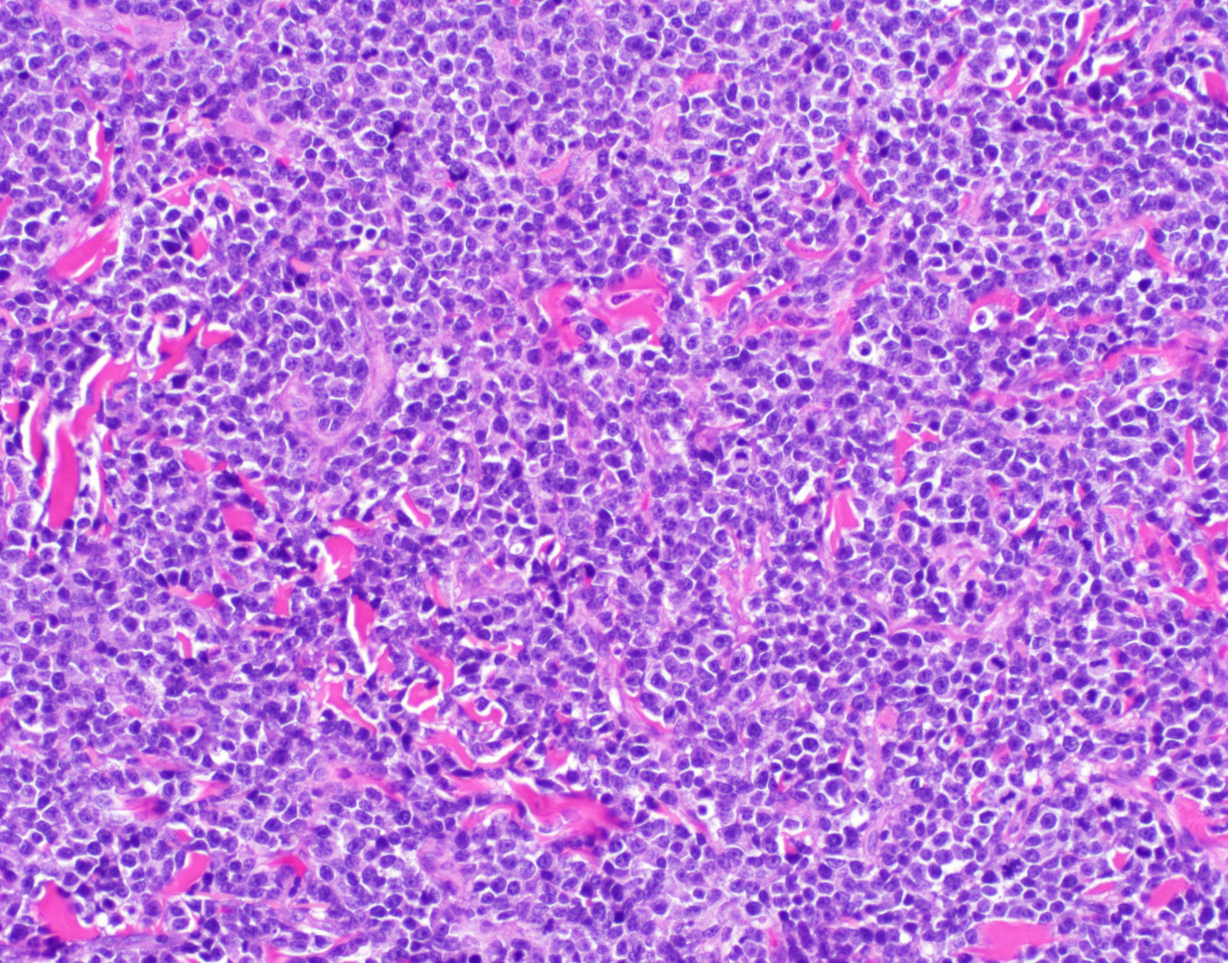
Hematoxylin and eosin stain of skin biopsy of cutaneous nodule from right upper leg The constituent cells show marked plasmacytoid cytomorphology on higher power with an infiltrative growth pattern (200x magnification).

**Figure 8:**
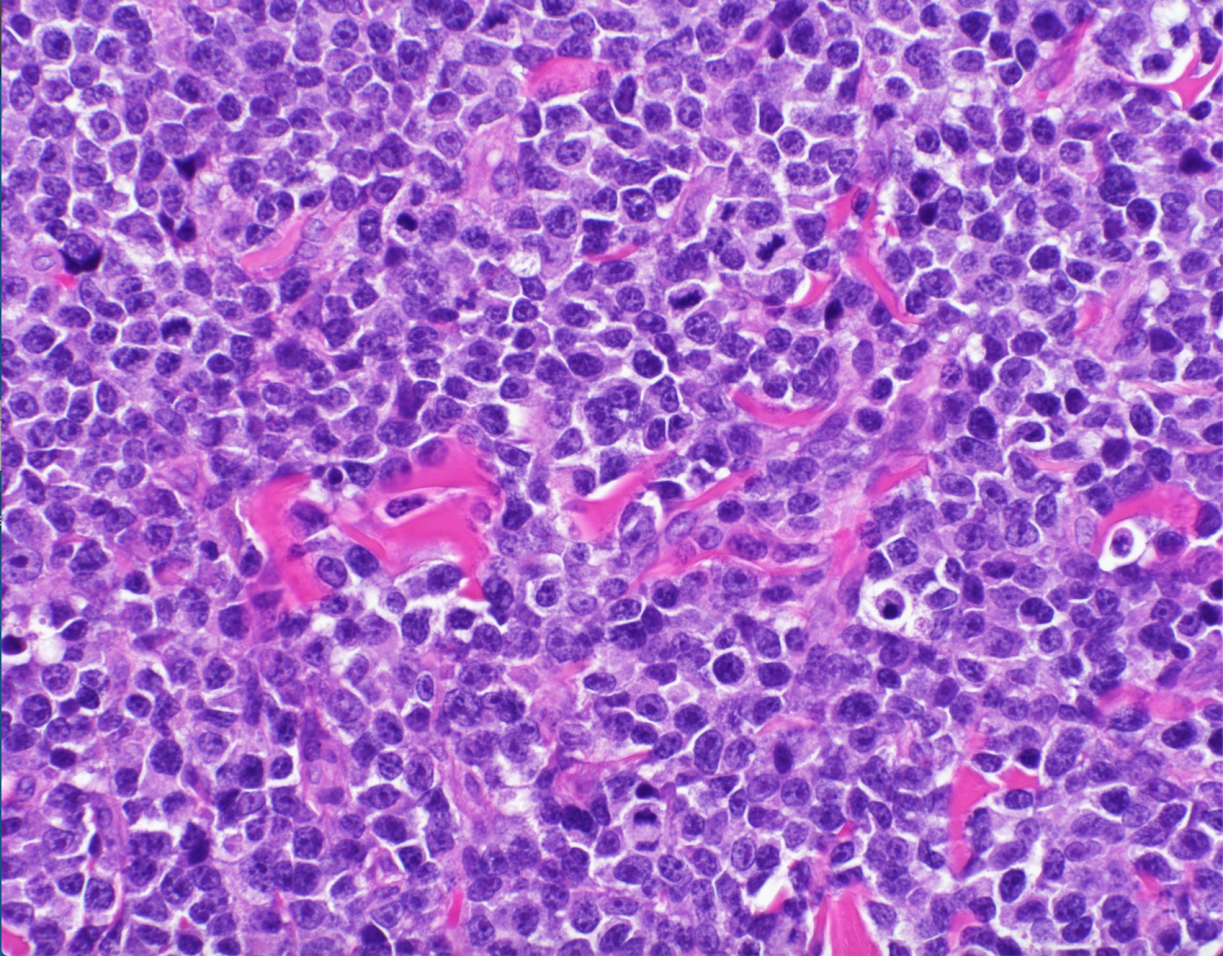
Hematoxylin and eosin stain of skin biopsy of cutaneous nodule from right upper leg High-grade nuclear features are present with hyperchromasia, angulation, and numerous atypical plasmacytoid cells captured in mitosis (400x magnification).

**Figure 9:**
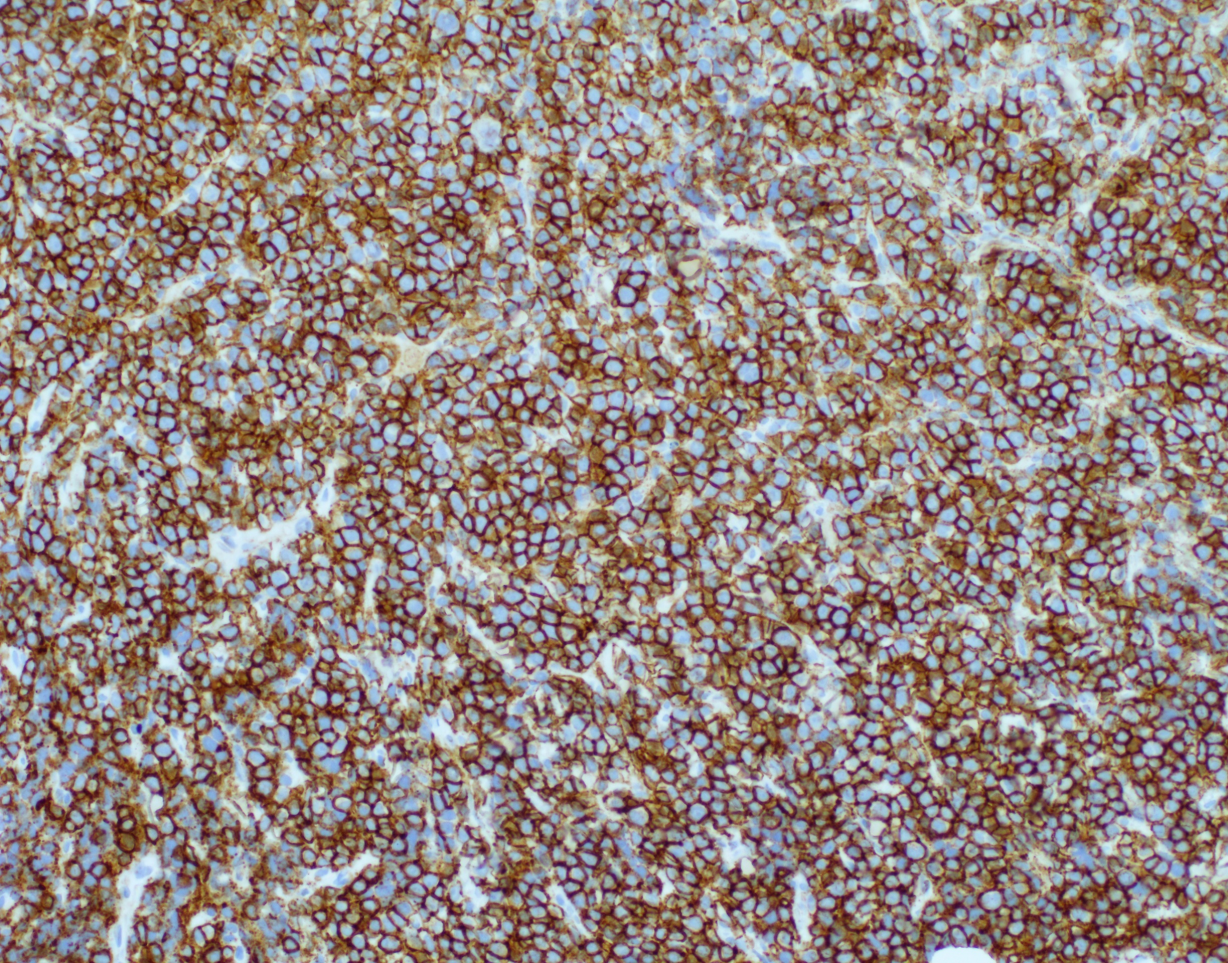
CD138 immunoperoxidase immunohistochemical stain of skin biopsy of cutaneous nodule from right upper leg CD138 shows strong immunolabeling, thus confirming plasma cell lineage (200x magnification).

**Figure 10:**
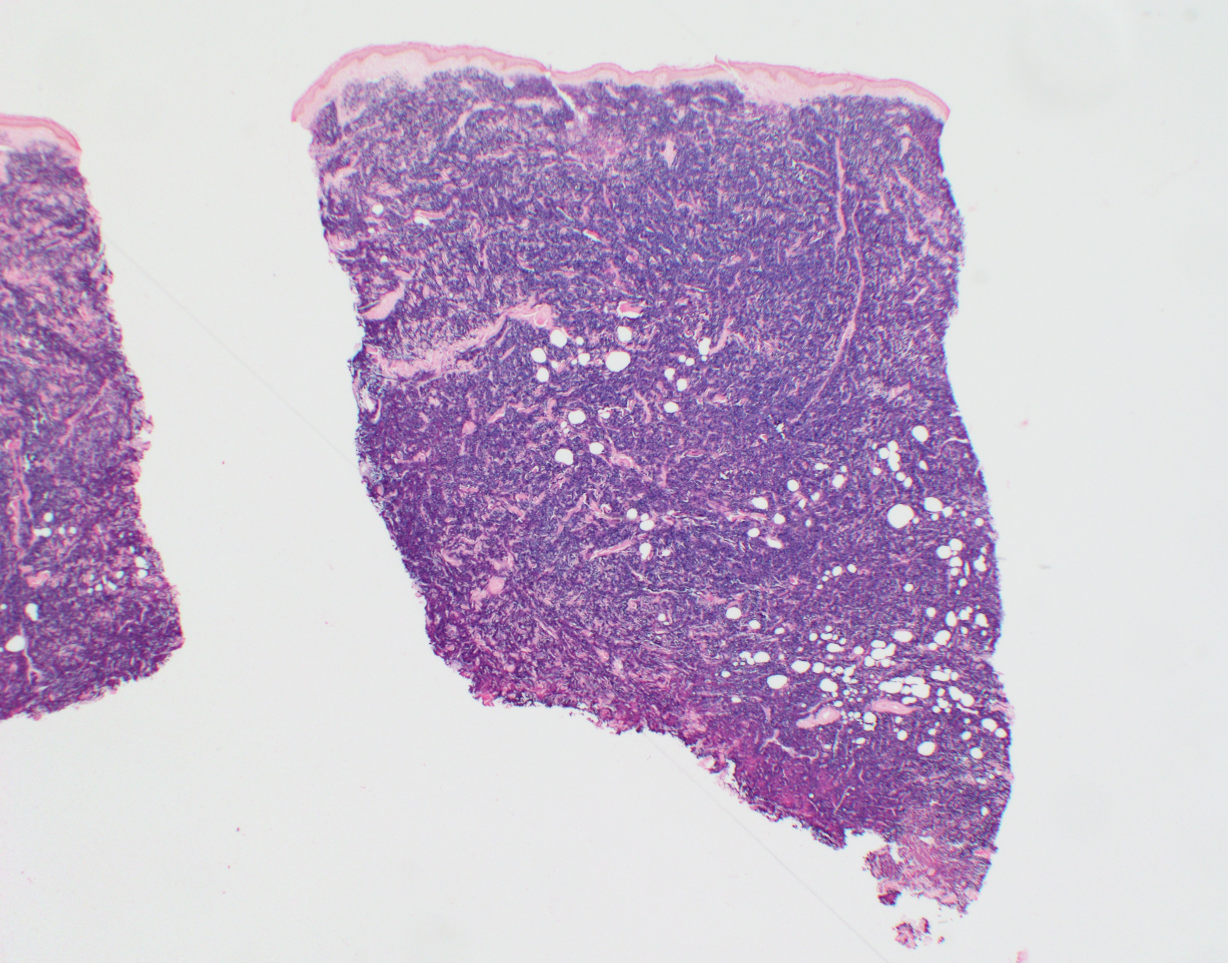
Kappa light chain immunoperoxidase immunohistochemical stain of skin biopsy of cutaneous nodule from right upper leg Chromogenic in-situ hybridization for kappa light chain shows diffuse positivity (20x magnification).

**Figure 11:**
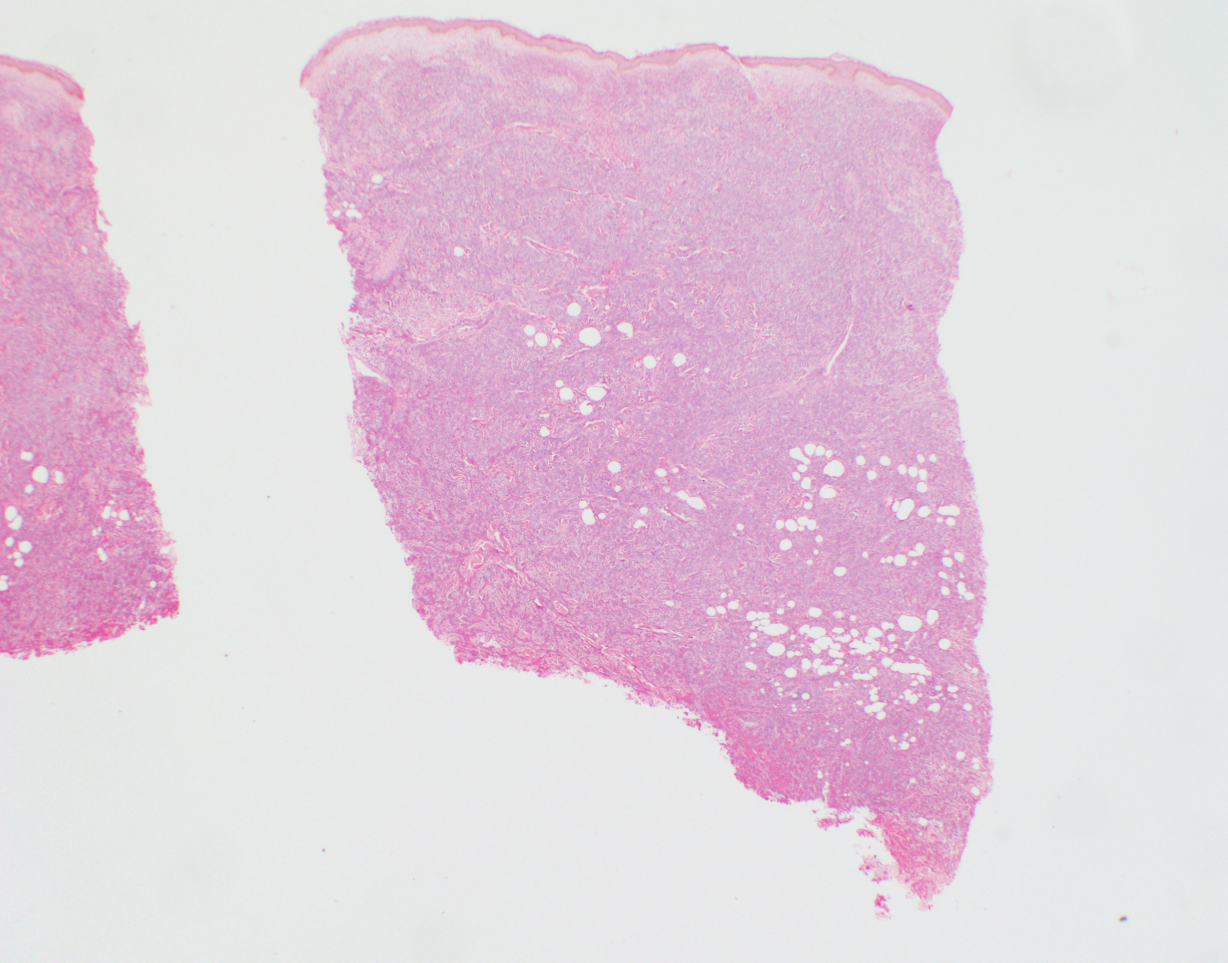
Lambda light chain immunoperoxidase immunohistochemical stain of skin biopsy of cutaneous nodule from right upper leg Chromogenic in-situ hybridization for lambda light chain reveals a lack of expression within the tumor cells of interest (20x magnification).

A punch biopsy for pathology and tissue culture (bacterial, fungal, and mycobacteria) was performed on the right upper leg on 11/11/2016. The biopsy showed sheets of plasmacytoid cells arranged in a nodular aggregate with pronounced atypical nuclei (Figures 5-8). Immunohistochemistry was performed with CD20 (B-lymphocyte antigen), PAX-5 (B-cell-specific activator protein), CD138 (syndecan-1, a plasma cell marker), and kappa/lambda chromogenic in situ hybridization. The cells of interest were negative for CD20 and PAX-5. Plasma cell lineage was confirmed with CD138 immunolabeling of the neoplastic cells (Figure 9), and clonality was demonstrated with kappa-light chain restriction (Figures 10-11). These results corresponded to previous characteristics of the patient’s myeloma. Tissue cultures were negative.

Correlation of the history, clinical presentation, and histopathology/immunohistochemical studies confirmed the diagnosis of plasma cell leukemia. The patient’s progressive vision loss and back pain was attributable to central nervous system involvement of his plasma cell leukemia. The patient received dartumumab (monoclonal CD38-inhibitor) for his systemic disease. He also received intrathecal cytarabine before and after radiation to treat his central nervous system relapse.

The patient was later admitted for failure to thrive and shortness of breath, with concern of a possible lung infection [3]. He was then transferred to hospice care and succumbed to his underlying disease and sepsis. The patient died at day 75 post-transplant, 25 days after his diagnosis of leukemia cutis.

## Discussion

The patient described in this report had leukemia cutis associated with secondary plasma cell leukemia. Leukemia cutis is an infiltration of the skin by malignant leukocytes or their precursors. Leukemia cutis often presents in association with systemic leukemia, but can also be a precocious manifestation, rarely appearing prior to leukemic cells appearing in peripheral blood or bone marrow. It is associated with a poor prognosis with life expectancy of less than 12 months after diagnosis [2, 4-5]. Leukemia cutis is most commonly seen in patients with acute myelogenous leukemia, but also occurs in other hematologic malignancies [4].

Plasma cell leukemia – an aggressive, uncommon variant of leukemia – represents only two percent of plasma cell neoplasms. Plasma cell leukemia is defined by greater than 2 × 10^9^ plasma cells per liter in a peripheral blood smear, or more than 20% of peripheral blood cells [1]. There are two forms of plasma cell leukemia: primary (de novo in the absence of multiple myeloma) and secondary (occurring within the context of multiple myeloma) [6]. When plasma cell leukemia develops within the context of multiple myeloma, as in our patient, it is considered an aggressive variant of multiple myeloma [2]. In patients with secondary leukemia cutis associated with multiple myeloma, the immunophenotype of the cutaneous infiltrate typically matches that of the patient’s myeloma [5].

The lesions of leukemia cutis in patients with plasma cell leukemia have been referred to as cutaneous plasmacytomas or extramedullary plasmacytomas of the skin [6]. Plasmacytomas are clonal proliferations of plasma cells in tissue that may be extraosseous or extramedullary [2]. Clinically, cutaneous plasmacytomas present as solitary or multiple slowly growing violaceous or purpuric dermal or subcutaneous nodules [6].

Pathergy is the development of a cutaneous condition at a prior site of trauma. Pathergy may trigger the development of plasma cell leukemia cutis. One report describes a 62-year-old man with plasma cell leukemia cutis who developed plasma cell leukemia cutis at venipuncture sites where needles and catheters had been inserted [7]. Interestingly, cutaneous leukemic infiltrations have been observed to occur in locally immunocompromised cutaneous areas, secondary to burns, herpes zoster viral infections, intramuscular injections, recent surgeries, and trauma [4, 8].

The evaluation of new skin lesions in leukemic patients should always include a skin biopsy for pathology and tissue cultures. Light microscopy should be performed, along with immunohistochemical stains or immunophenotyping when indicated. Bacterial, fungal, and mycobacterial cultures should also be obtained to evaluate for possible infectious etiology [9]. Our patient’s lesions were a cutaneous manifestation of his underlying plasma cell leukemia. However, we also considered infectious causes in the differential diagnosis of his subcutaneous nodules, as the patient was an immunocompromised individual.

The therapeutic management of plasma cell leukemia cutis is to target the underlying systemic disease. This may be accomplished by systemic chemotherapy in combination with local therapy such as radiation or surgery [7, 9]. Our patient received dartumumab (monoclonal CD38-inhibitor) for his systemic disease, with one dose of intrathecal cytarabine prior to and following radiation for his central nervous system relapse. However, he succumbed to his plasma cell leukemia-myeloma 25 days after developing leukemia cutis.

## Conclusions

Leukemia cutis associated with plasma cell leukemia, as confirmed by biopsy in our patient, is rare. Plasma cell leukemia may occur de novo or in association with multiple myeloma. Leukemia cutis in patients with plasma cell leukemia is an extraordinarily uncommon clinical presentation and associated with a very poor prognosis. New cutaneous lesions in a patient with plasma cell leukemia should be regarded with great concern and biopsied promptly to establish the definitive diagnosis. Treatment for plasma cell leukemia cutis should be directed at treating the underlying systemic disease.
